# In vitro visualization and characterization of wild type and mutant IDH homo- and heterodimers using Bimolecular Fluorescence Complementation

**DOI:** 10.17980/2016.311

**Published:** 2016-07-09

**Authors:** Gemma L. Robinson, Beatrice Philip, Matthew R. Guthrie, James E. Cox, James P. Robinson, Matthew W. VanBrocklin, Sheri L. Holmen

**Affiliations:** 1 Department of Surgery, University of Utah Health Sciences Center, Salt Lake City, UT, 84112, USA; 2 Metabolomics Core Research Facility, University of Utah School of Medicine, Salt Lake City, UT, 84112, USA; 3 The Hormel Institute, University of Minnesota, Austin, MN, 55912, USA; 4 Huntsman Cancer Institute, Salt Lake City, UT, 84112, USA

**Keywords:** malignant glioma, isocitrate dehydrogenase (IDH), bimolecular fluorescence complementation (BiFC), fluorescent imaging, protein-protein interactions

## Abstract

Mutations in the metabolic enzyme *isocitrate dehydrogenase* (IDH) were recently found in ~80% of WHO grade II–III gliomas and secondary glioblastomas. These mutations reduce the enzyme’s ability to convert isocitrate to α-ketoglutarate and, instead, confer a novel gain-of-function resulting in the conversion of α-ketoglutarate to 2-hydroxglutarate (2-HG). However, IDH mutations exist in a heterozygous state such that a functional wild type allele is retained. Recent data suggest that the ability of mutant IDH1, but not mutant IDH2, to produce 2-HG is dependent on the activity of the retained wild type allele. In this study, we aimed to further our understanding of the interaction and function of wild type and mutant IDH heterodimers utilizing Bimolecular Fluorescence Complementation (BiFC). Dimerization of wild type and mutant IDH monomers conjugated to the N- and C-terminus of Venus protein, respectively, is directly proportional to the amount of fluorescence emitted and can be used as an approach to visualize and assess IDH dimerization. Thus, we utilized this method to visualize IDH homo- and heterodimers and to examine their cellular physiology based on subcellular localization, NADPH production, and 2-HG levels. Our results demonstrate that wild type and mutant IDH1 or IDH2 heterodimers display similar physiological characteristics to that of mutant IDH1 or IDH2 homodimers with the exception of their ability to generate NADPH. IDH1 heterodimers consistently generate NADPH whereas IDH2 heterodimers do not. However, the presence of mutant IDH1 or IDH2 in homo- or heterodimer configurations consistently generates equivalent levels of 2-HG. Our data suggest that the wild type protein is not required for the generation of 2-HG.

## Introduction

Diffuse gliomas are the most common primary brain tumor in adults and are associated with a poor prognosis (1). The majority of gliomas are astrocytic while the remainder are oligodendroglial tumors (2). Diffuse gliomas are further sub-categorized according to their grade; low grade (grade II), anaplastic (grade III) and glioblastoma (GBM, grade IV) (2) GBM is the most common and aggressive glioma (3). It is also the most fatal (3, 4). Despite major improvements in imaging, radiation, and surgery, the prognosis for patients with this disease is still poor (5). A better understanding of the biology of these gliomas will guide the development of new therapies to improve survival and reduce morbidity.

Recently, whole exome sequencing of patient GBM samples identified a heterozygous missense mutation in the metabolic enzyme *isocitrate dehydrogenase* (IDH), which was found to be present in 70–80% of WHO grade II–III gliomas, namely astrocytomas and oligodendrogliomas, and secondary GBM (6, 7). While the discovery of mutant IDH in a high percentage of gliomas is suggestive of a role for this genetic alteration in the etiology of GBM, this has yet to be validated. IDH proteins function to generate reduced nicotinamide adenine dinucleotide phosphate (NADPH) from NADP^+^ by catalyzing the oxidative decarboxylation of isocitrate to α-ketoglutarate (αKG). In malignant gliomas, the mutations occur in either cytosolic IDH1 or mitochondrial IDH2. The most common point mutations occur at R132H and R172K in IDH1 and IDH2, respectively, both of which are embedded within the active site of the enzyme (6). These mutations inhibit the enzymes ability to convert isocitrate to α-ketoglutarate and instead confer a gain-of-function resulting in the conversion of α-ketoglutarate to 2-hydroxyglutarate (2-HG) (8). Key to this finding is that elevated levels of 2-HG are a specific biomarker of secondary GBMs harboring IDH mutations (9, 10).

Under physiological conditions, both IDH1 and IDH2 exist as homodimers in the cytosol and mitochondria, respectively (11). Dimerization is mediated by interactions between the two clasp domains of each IDH protein and dimeric IDH contains two active sites each composed of amino acid residues from both subunits (11). Thus, the dimerization of IDH is essential for its enzymatic role within the cell. Since IDH mutations exist as heterozygous mutations in glioma cells it is assumed that a combination of wild type and mutant homo- and heterodimers co-exist. The role of mutant IDH1 homodimers has been studied extensively by analyzing the overexpression of mutant IDH *in vitro* (8, 12, 13). Recent data suggest, however, that the ability of cytosolic mutant IDH1 to produce 2-HG is dependent on the activity of the retained wild type allele (14). Ward *et al.,* reported that cytosolic mutant IDH1 generated significantly elevated levels of intracellular 2-HG only when an equivalent level of wild type IDH1 was co-expressed (14). Mitochondrially expressed wild type IDH2, however, was not essential for mutant IDH2 to elevate intracellular levels of 2-HG (14). Furthermore, it was observed that brain-specific mutant IDH1 conditional knock-in mice were perinatal lethal; an effect proposed to relate to extensive 2-HG production (15). 2-HG is structurally similar to α-ketoglutarate (α-KG) and thus can inhibit α-KG-dependent enzymes resulting in extensive epigenetic alterations [reviewed in (16)]. As a result, the aberrant role of mutant IDH is proposed to directly correlate with the amount of 2-HG produced. However, the ability of 2-HG accumulation to directly alter gene expression has not been evaluated. The stoichiometry of IDH1 and 2 homo- and heterodimers, and their correlation with 2-HG levels, has also not been determined. Understanding the biology of mutant IDH is essential to deciphering the therapeutic benefits of targeting these mutations in cancer.

In this study, we assessed the ability of wild type IDH1 and 2 to dimerize and regulate the aberrant activity of mutant IDH1 and 2, respectively. For this, we employed a Bimolecular Fluorescence Complementation (BiFC) based approach that permitted the *in vitro* visualization and characterization of IDH homo- and heterodimers. We assessed wild type and mutant homo- and heterodimer interactions, protein expression, subcellular localization, enzymatic activity and 2-HG production. We observed that mutant–mutant homodimers can generate 2-HG as efficiently as wild type–mutant heterodimers demonstrating that the wild type protein is not required for the generation of 2-HG.

## Materials and Methods

### BiFC construct cloning

Asymmetric PCR was used to create IDH-Venus (BiFC) fusion proteins. IDH construct DNA was isolated by PCR amplification from retroviral vectors containing wild type and mutant IDH1 or IDH2. The N-terminal 172 amino acid residues of Venus (VN173) and C-terminal residues (155-238) of Venus (VC155) were fused to the N-terminus of IDH1 or the C-terminus of IDH2 with a ProTEV linker sequence inserted between Venus and the IDH constructs. VN173 and VC155 construct DNA, and VN173-RalBP1 and VC155-RalA, were kindly provided by Matthew VanBrocklin (Huntsman Cancer Institute). Primers used for asymmetric PCR are listed in Supplementary Table 1. PCR fusion-products were cloned into the pCR8/GW/Topo vector (Invitrogen) and gateway cloned into the destination vector pDEST12.2 using LR Clonase II as per the manufacturer’s specifications (Invitrogen).

### Cell Culture and Transfection

HEK293FT cells were cultured in Dulbecco’s Modified Eagle Medium (DMEM) supplemented with 10% fetal bovine serum and 0.5% gentamicin, and maintained at 37°C in a humidified atmosphere of 5% CO_2_. Transfections were performed using Lipofectamine 2000 (Invitrogen) as per the manufacturer’s instructions. Co-transfections were performed with the indicated combinations of Venus-IDH constructs using 0.25 μg of each construct (0.5 μg Total DNA). To confirm expression of individual constructs, 0.5 μg of each construct DNA was transfected alone. Cells were harvested and analyzed 18–20 h post-transfection.

### CRISPR/CAS9 gene targeting

CRISPR/CAS9 lentiviral vectors with a guide RNA (gRNA) targeting *IDH1*, developed by the mutation generation core at the University of Utah, were used to disrupt expression of the endogenous IDH1 gene in HEK293FT cells. Forty eight hours post infection with these lentiviral vectors, the cells were selected with 1.0 ug/ml puromycin. Individual clones were expanded and CRISPR/CAS9 mediated *IDH1* sequence modification was assessed using High Resolution Melt Analysis, immunoblot and sequencing.

### High resolution melt analysis (HRMA)

DNA from IDH1 knockout (KO) 293FT cell clones was isolated using the DNEasy blood and tissue kit (Qiagen). For the initial polymerase chain reaction, primers flanking the target site were used to amplify the genomic region in a 10 μl reaction containing 60 ng of DNA, 4 μl of 1X LightScanner Master mix (Idaho Technologies), 0.2 μM of each dNTP, and 0.2 μM of each forward and reverse primer (Supplementary Table 1). Samples were amplified for 50 cycles (94° C for initial denaturation for 3 min followed by 94° C for 30 s, 70° C for 20 s). HRMA was performed at temperatures between 65–95° C and data was collected on a LightScanner (Idaho Technologies). Melt curves were generated by measuring the decrease in fluorescence of LCGreen plus dye and analyzed using LightScanner Call-IT software.

### Whole Cell Imaging

Whole cell *in vitro* fluorescent complementation was confirmed using a Nikon Eclipse Ti inverted fluorescence microscope with perfect focus. Images were processed using Nikon’s Elements Software and images were analyzed for percentage BiFC fluorescence using CellProfiler^™^ Software. Control combinations for BiFC constructs (S2) were imaged using an Olympus CKX41 inverted fluorescent microscope. Images were captured using a QICAM Fast 1394 digital camera (QImaging) and processed using QCapturePro software (QImaging). All images are 20x magnification unless stated otherwise.

### Immunoblotting

Whole cell lysates were collected using SDS-lysis buffer (200 mM TRIS/HCl (pH 6.8), 2% SDS, 0.1M DTT, 10% Glycerol, 0.1% bromophenol Blue). Proteins were separated on 4–20% tris-glycine polyacrylamide gels (Invitrogen) and transferred to nitrocellulose. Blots were probed with the indicated primary and secondary antibodies, and detected using Amersham ECL Western Blotting Detection Reagents (GE Healthcare). Primary antibodies used were rabbit anti-IDH1 (Cell Signaling #8137), mouse anti-IDH1 R132H (Dianova #DIA-H09), rabbit anti-IDH2 (Santa Cruz Biotech #SC134923), rat anti-IDH2 R172K (MBL #D328-3), mouse anti-GAPDH (Millipore #MAB374), mouse anti-HA (Covance #3956) and mouse anti-Cas9 (clone 7A9-3A3; Cell Signaling #14697). HRP-conjugated secondary antibodies used were: anti-mouse IgG (Cell Signaling), anti-Rabbit IgG (Cell Signaling) and anti-rat IgG (Cell Signaling).

### Subcellular Localization

HEK293FT cells were seeded onto poly-L-lysine coated coverslips and transfected with the indicated BiFC-constructs. Cells were counterstained with Hoechst (Invitrogen), MitoTracker Red (Invitrogen) and/or CellTracker Orange (Invitrogen) as per the manufacturer’s instructions. Cells were then washed in ice cold PBS and fixed for 10 min with 3.8 % formaldehyde. Coverslips were transferred to poly-L-lysine coated microscope slides and cells were imaged using a Nikon Eclipse 90i Fluorescence microscope using 60x oil immersion. Images were analyzed using ImageJ software.

### IDH Activity Assay

IDH activity was measured using an Isocitrate Dehydrogenase Activity Colorimetric Assay Kit (BioVision) as per the manufacturer’s protocol. Briefly, transfected HEK293FT cells were washed in ice cold PBS and homogenized in 200 μl IDH assay buffer. 50 μl of homogenized sample was transferred to one well of a 96-well plate, in triplicate, and mixed with 50 μl of assay reaction mix. The plate was incubated for 3 min at 37 °C and read at OD450 every 5 min for 2 h on a Synergy HT Multi-Detection Microplate Reader (Bio-Tek). Measurements were used to calculate IDH activity based on the generation of NADPH. For IDH competition assays, HEK293FT cells were co-transfected with BiFC-conjugated constructs along with increasing concentrations of HA-tagged wild type or mutant IDH1/2 and assayed for IDH activity as described above.

### 2-HG Measurements

Two 10 cm plates of HEK293FT cells were transfected 48 h prior to harvesting with the indicated BiFC constructs. Cells were washed in ice cold PBS and snap frozen at −80 °C. For mass spectrometry analysis, cell pellets were resuspended in ice-cold 90% methanol containing 1 μg of the internal standard d_4_-succinate. GC-MS analysis was conducted using a Waters GCT Premier mass spectrometer and data was collected using Waters Masslynx Software. The raw area for each analyte was normalized based on the response factor calculated for the added internal standard.

## Results

### Generation of IDH-Venus constructs for BiFC Analysis

**Figure 1:**
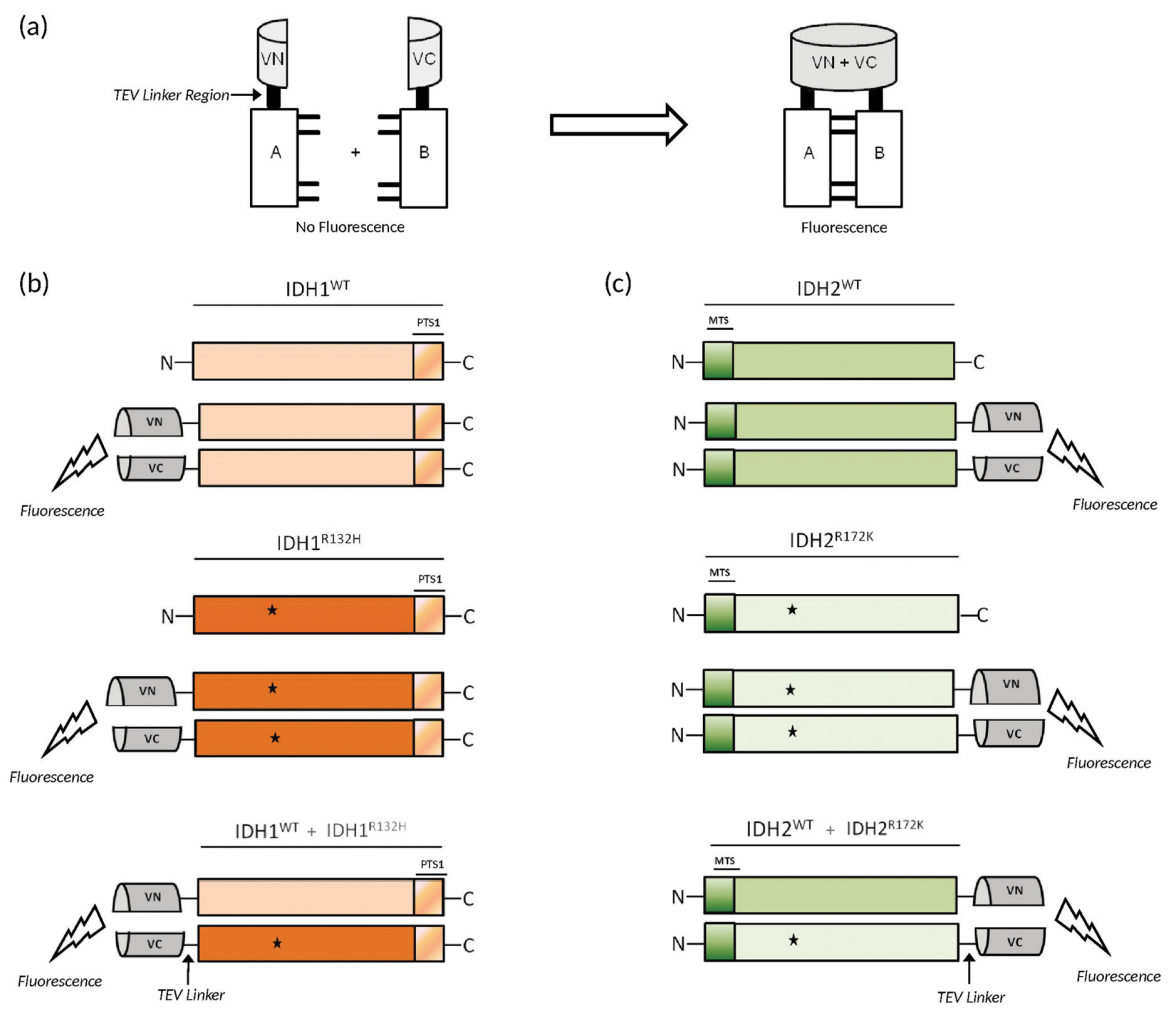
Dimerization schematics for wild type and mutant IDH1 and IDH2 homo- and heterodimers conjugated to their respective Venus constructs (a) Schematic to demonstrate the principles of BiFC when the N- and C-termini of Venus protein are fused to the N-termini of proteins A and B, respectively. Protein-Protein interaction permits formation of a Venus-based bimolecular fluorescence complex resulting in fluorescence. Image modified from [17]. (b) Schematics to show IDH1 wild type and mutant homo- and heterodimers. (c) Schematics to show IDH2 wild type and mutant homo- and heterodimers. Dimerization between IDH monomers permits Venus complex formation via association of the N-terminal (VN) and C-terminal (VC) subunits leading to fluorescence. PTS1; peroxisomal targeting sequence type 1, MTS; mitochondrial targeting sequence, VN; N-terminal Venus (amino acids 1-173), VC; C-terminal Venus (amino acids 155-238). The Tobacco Etch Virus (TEV) linker region is located between IDH and the Venus construct. ★ indicates the R132H and R172K mutations for IDH1 and IDH2, respectively.

To visualize and examine the interaction between wild type and mutant IDH homo- and heterodimers, we employed the protein-protein interaction assay Bimolecular Fluorescence Complementation (BiFC) (17). This approach has been used extensively to study protein-protein interactions in living cells and is based on the formation of a fluorescent complex though the association of two fragments of a fluorescent reporter protein (Venus) fused to two proteins of interest (17–21). An interaction between the proteins facilitates association between the two Venus fragments to produce a bimolecular fluorescent complex (Figure 1a). Using this approach, we generated IDH-Venus fusion proteins such that expression of the fluorescent complex occurs when analogous IDH homo- and heterodimers form (Figure 1b–c). In addition, a linker region (Tobacco Etch Virus [TEV]) was inserted between IDH and Venus to ensure proper function of IDH enzymes (Figure 1a–c). Homo- and heterodimerization of wild type and mutant IDH1 or 2 monomers generates a fluorescent complex only under conditions of complementary binding, and not with related or unrelated proteins expressing Venus constructs (Supplementary Figure 1). Since IDH1 has a C-terminal peroxisomal targeting sequence, the N-terminal 172 amino acid residues of Venus (VN) and the C-terminal 155-238 residues of Venus (VC) were fused to the N-terminus of wild type and mutant IDH1 (Figure 1b). IDH2, however, has an N-terminal mitochondrial targeting sequence and, thus, the N- and C-terminal fragments of Venus were fused to the C-termini of IDH2 (Figure 1c).

### Protein Expression Levels of IDH-Venus Constructs

**Figure 2:**
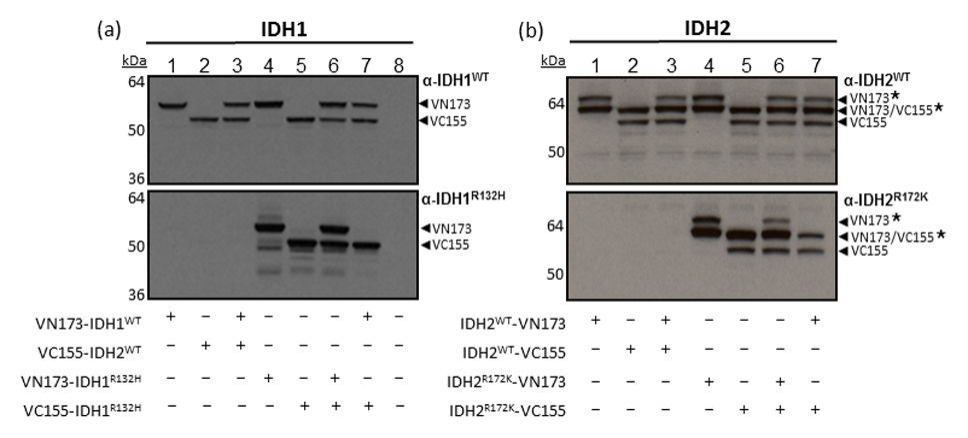
IDH1 and IDH2 homo- and heterodimer BiFC constructs are expressed at the protein level and demonstrate size differences consistent with fusion to their respective halves of Venus HEK293FT cells were transfected with the indicated IDH1 (a) or IDH2 (b) BiFC constructs and assessed for protein expression 24h post-transfection using IDH1 or 2 wild-type and mutant specific antibodies. * indicates immature form of each IDH2 fusion protein.

To confirm expression of Venus-conjugated IDH1 and 2 constructs, HEK293FT cells were transiently transfected with the indicated IDH-Venus constructs and assessed for protein expression by immunoblotting (Figure 2a–b). Wild type and mutant IDH1 conjugated to the N-terminal half of Venus (VN173) show protein expression levels consistent with that of a VN173-IDH1 fusion protein (Figure 2a, lanes 1, 3, 4, 6 and 7). Wild type and mutant IDH1 conjugated to the C-terminal half of Venus protein (VC155) show protein expression levels consistent with that of a VC155-IDH1 fusion protein (Figure 2a, lanes 2, 3, 5, 6 and 7). HEK293FT cells expressing one version of each Venus-IDH1 fusion construct, as in the case of homo- and heterodimers, can be distinguished by these inherent size differences (Figure 2a, lanes 3, 6 and 7). Exogenous expression of wild type or R132H-mutant IDH1 homo- and heterodimers can be distinguished using an IDH1-R132H mutant specific antibody (Figure 2a, lower panel). Cellular expression of Venus-IDH1 fusion genes is equivalent across each combination demonstrating near equal expression of each construct. Similar to IDH1, wild type and mutant IDH2 conjugated to the N- (VN173) or C-termini (VC155) of Venus demonstrate protein expression levels consistent with that of IDH2-Venus fusion proteins (Figure 2b, lanes 1–7). For IDH2-Venus protein monomer expression, we observed the expression of two protein bands (Figure 2b, lanes 1, 2, 4 and 5). Endogenous expression of IDH2 in HEK293FT cells also appears to show the expression of two protein bands (Figure 2b), which we propose distinguishes between a mature mitochondrially expressed IDH2 and that of immature IDH2, which still contains its mitochondrial-targeting sequence. Exogenous expression of wild type or R172K-mutant IDH2 homo- and heterodimers can be distinguished using an IDH2-R172K mutant specific antibody (Figure 2b, lower panel). Cellular expression of IDH2-Venus fusion genes is equivalent across each combination demonstrating near equal expression of each construct. Together, these data demonstrate that IDH1 and 2 Venus-fusion proteins are expressed at the protein level and demonstrate size differences consistent with fusion to their respective halves of Venus (Figure 2a–b).

### In Vitro Visualization of IDH Venus-fusion Homo- and Heterodimers

**Figure 3:**
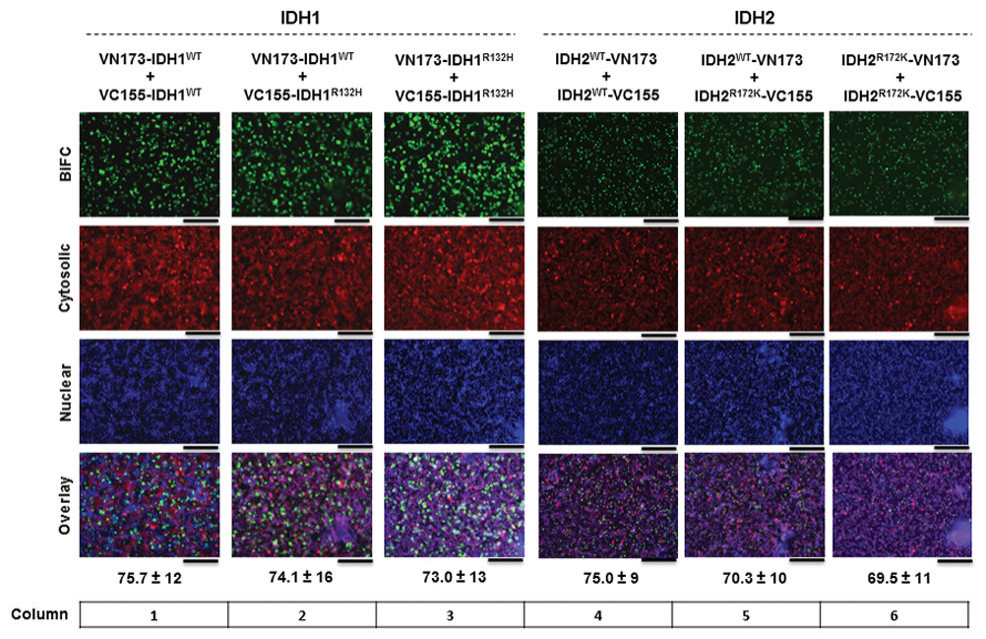
Visualization of wild-type and mutant IDH1 and 2 homo- and heterodimer formation *in vitro* using BiFC analysis HEK293FT cells were co-transfected with the indicated IDH1 or 2 wild type and mutant BiFC fluorescent reporter constructs, and counterstained with nuclear and cytosolic markers to confirm complex formation. Images are representative of three independent experiments 24 h after transfection. Values below the images represent % BiFC positive cells ± SEM from 3 independent experiments. Scale bars represent 50 μM.

Under physiological conditions, wild type IDH1 or IDH2 form homodimers (11). To visualize IDH homodimerization using our BiFC-based approach, HEK293FT cells were co-transfected with wild type VN173- and VC155-conjugated IDH fluorescent reporter monomers (Figure 3, columns 1 and 4). *In vitro* cellular fluorescence was assessed 24 h post-transfection using fluorescence microscopy and cells were counterstained with nuclear and cytosolic markers to quantitate the amount of cellular BiFC fluorescence (Figure 3). The dimerization of wild type IDH-Venus monomers demonstrated greater than 75% fluorescent complementation for both IDH1 and IDH2 (Figure 3, columns 1 and 4). To assess dimerization of IDH1 wild type and mutant heterodimers, or mutant IDH1 homodimers, cells were transfected with the indicated Venus-IDH1 fusion constructs and assessed for cellular fluorescence 24 h post-transfection (Figure 3, columns 2–3). Similar to wild type IDH1 homodimer fluorescence, BiFC expression of wild type and mutant IDH1 heterodimers and mutant IDH1 homodimers demonstrates greater than 70% fluorescence (Figure 3, columns 2–3). Together, these data indicate that wild type and mutant IDH monomers are able to form heterodimers, under physiological conditions, as efficiently as wild type IDH1 homodimers.

To assess dimerization of IDH2 wild type and mutant heterodimers, or mutant IDH2 homodimers, cells were transfected with the indicated IDH2-Venus fusion constructs and assessed for cellular fluorescence 24 h post-transfection (Figure 3, columns 5–6). BiFC fluorescence was observed to be greater than 70% for both wild type and mutant IDH2 heterodimers and mutant IDH2 homodimers (Figure 3, columns 5–6). Together, these data show that IDH2-Venus dimers form efficiently between wild type or mutant IDH2 homodimers and wild type and mutant heterodimers.

### Subcellular Localization of IDH BiFC Constructs

**Figure 4:**
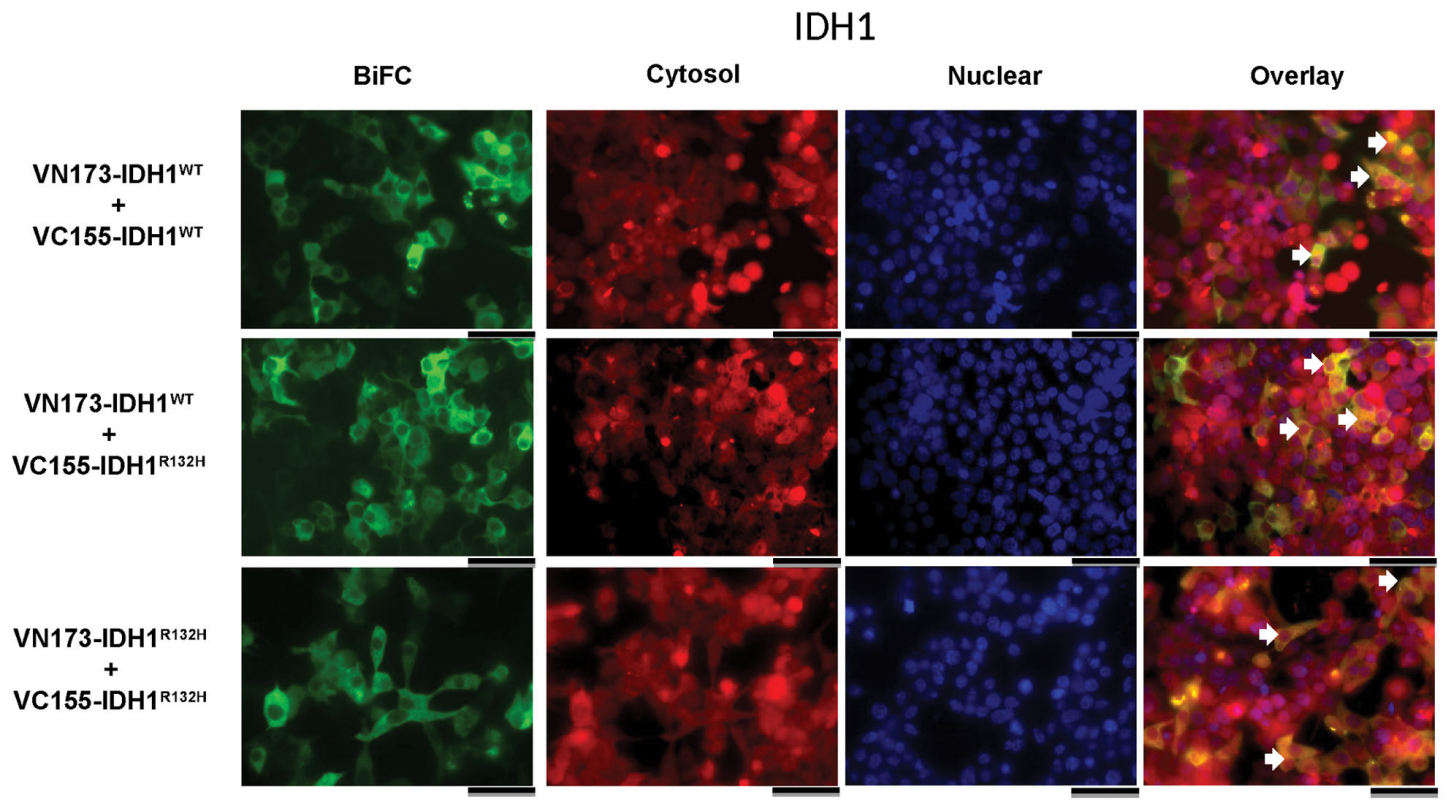

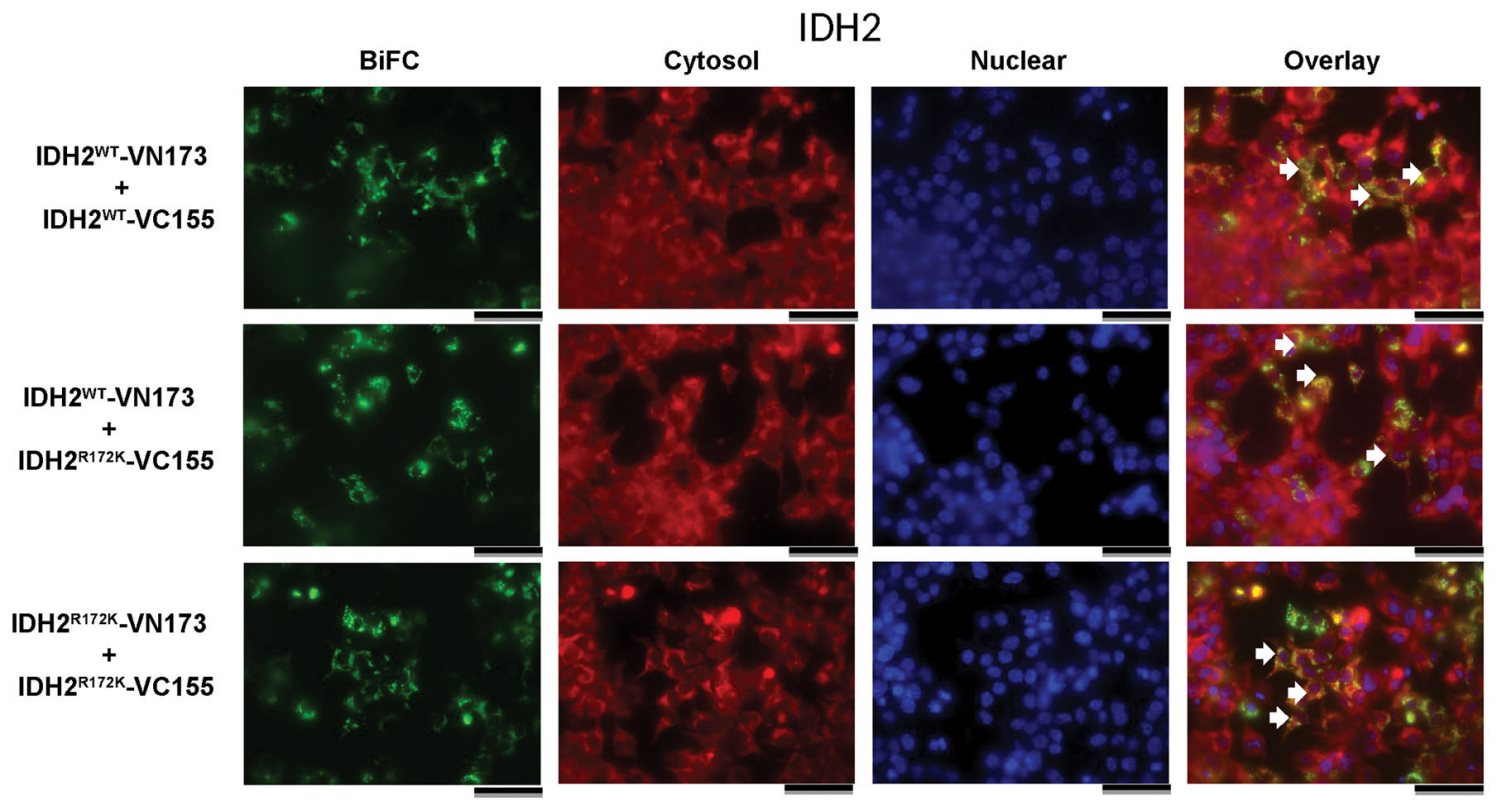
BiFC conjugated IDH1 and 2 homo- and heterodimers reside in the cytosol and mitochondria, respectively HEK293FT cells were transfected with the indicated BiFC constructs and co-stained with CellTracker^™^ Orange (IDH1) or MitoTracker^™^ Red (IDH2) and Hoechst. (a) IDH1 dimer BiFC fluorescence displays co-localization with CellTracker^™^ Orange indicative of global cytosolic expression. (b) IDH2 dimer BiFC fluorescence displays co-localization with MitoTracker^™^ Red consistent with IDH2 localization to the mitochondria. Images are representative of three independent experiments. White arrows indicate the co-localization of BiFC expression with cytosol (a) or mitochondria (b), respectively. Scale bars represent 50 μM.

Under physiological conditions, IDH1 is localized to the cytosol and IDH2 is localized to the mitochondria. To confirm subcellular localization of IDH1 and IDH2 Venus fusion proteins, HEK293FT cells were co-transfected with the indicated IDH BiFC constructs and co-stained with either a cytosolic or mitochondrial fluorescent marker for assessment of IDH1 and IDH2 localization, respectively (Figure 4a–b). Nuclear staining demonstrates large, spherical nuclei consistent with viable cells (Figure 4a–b, ‘Nuclear’). Wild type or mutant IDH1 homodimers display BiFC fluorescence consistent with the staining observed with CellTracker^™^ Orange (Figure 4a, ‘Cytosol’), which is indicative of global cytosolic expression (Figure 4a, ‘Overlay’, arrows). Wild type and mutant IDH1 heterodimers also demonstrate staining consistent with that of wild type or mutant homodimers suggesting that dimer localization is similar between different IDH1 dimer combinations (Figure 4a). Unlike the fluorescence observed with IDH1 dimers, IDH2 homo- and heterodimer BiFC expression demonstrates punctate cellular fluorescence (Figure 4b, ‘BiFC’). This is consistent with Mitotracker^™^ red localization (Figure 4b, ‘Mitochondria’), which is indicative of localized mitochondrial expression (Figure 4b, ‘Overlay’, arrows). The localization of IDH2-Venus constructs to the mitochondria is similar between different IDH2 dimer combinations (Figure 4b). Together, these data demonstrate that IDH1 and IDH2 Venus-conjugated constructs are expressed in the cytosol and mitochondria, respectively (Figure 4).

### IDH-Venus Dimer Enzymatic Activity

**Figure 5:**
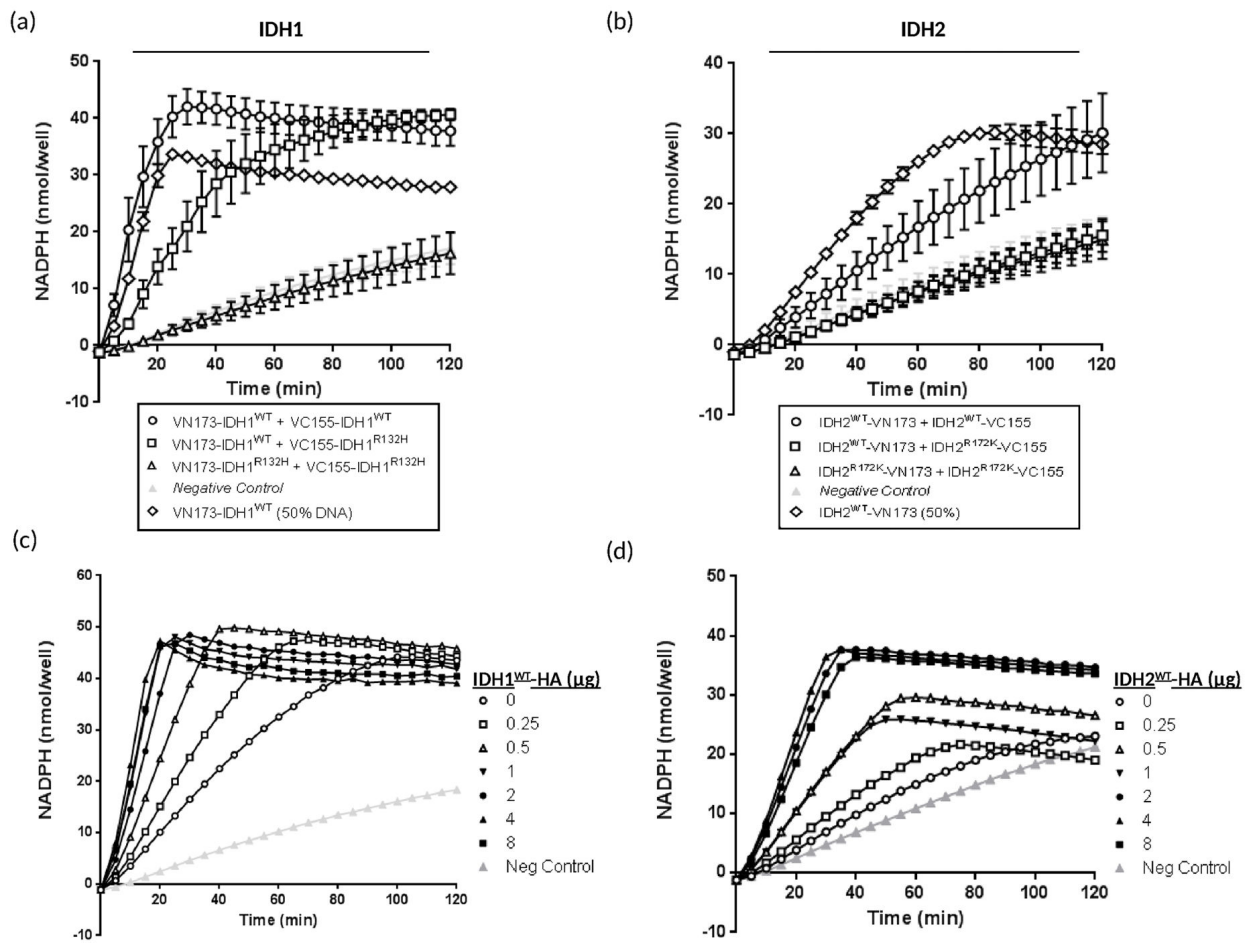
Wild type and mutant IDH1 and 2 homo- and heterodimers show differences in their ability to generate NADPH HEK293FT cells were transfected with the indicated IDH1 (a) or IDH2 (b) BiFC constructs or left untransfected (endogenous IDH control: gray lines) and assessed for their ability to generate NADPH in the presence of excess NADP^+^ and isocitrate using an Isocitrate Dehydrogenase Activity Colorimetric Assay Kit as per the manufacturer’s protocol. 0.25 μg of each DNA construct was transfected in a 24-well plate format (0.5 μg total). For 50% DNA samples, 0.25 μg of each single DNA construct was transfected. (c) 0.25 μg of each of VN173-IDH1^WT^ and VC155-IDH1^R132H^ were co-transfected into HEK293FT cells along with the indicated increasing concentration of IDH1^WT^-HA. Cells were assessed for their ability to generate NADPH using an Isocitrate Dehydrogenase Activity Colorimetric Assay Kit as per the manufacturer’s protocol. (d) 0.25 μg of each of IDH2^WT^-VN173 and IDH2^R172K^-VC155 were co-transfected into HEK293FT cells along with the indicated increasing concentration of IDH2^WT^-HA. Cells were assessed for their ability to generate NADPH using an Isocitrate Dehydrogenase Activity Colorimetric Assay Kit as per the manufacturer’s protocol. Data is representative of at least 3 independent experiments.

The physiological role of IDH is to convert isocitrate to α-ketoglutarate with the concomitant production of NADPH (11). The mutation of IDH1 or 2 at residues 132 and 172, respectively renders the enzyme inactive by altering the structure of the active site and thus inhibiting its normal cellular function (6). Thus, rather than generating NADPH, mutant IDH1 and 2 consume NADPH to enable the generation of 2-HG (8). To determine the ability of wild type and mutant IDH heterodimers to generate NADPH, we compared the levels of NADPH produced by wild type and mutant IDH heterodimers to that of wild type or mutant IDH homodimers (Figure 5a–b). Wild type IDH1 homodimers display significantly higher levels of NADPH production, in the presence of isocitrate, compared to that of mutant IDH1 homodimers, which exhibit NADPH levels similar to that of endogenous negative control levels (Figure 5a). Thus, mutant IDH1 homodimers are inactive with respect to their ability to generate NADPH. Interestingly, we observed that wild type and mutant IDH1 heterodimers could generate significantly higher levels of NADPH compared to mutant IDH1 homodimers (Figure 5a) and that this activity did not correlate solely with the presence of 50% wild type IDH1 expression that would exist in the IDH1 heterodimer combination (Figure 5a). Thus, wild type and mutant IDH1 heterodimers are able to generate NADPH while mutant IDH1 homodimers are inactive with respect to their ability to generate NADPH.

To assess the ability of IDH2-Venus dimers to generate NADPH, we measured NADPH levels in cells transfected with the indicated IDH2-Venus dimer combinations in the presence of isocitrate (Figure 5b). Similar to IDH1, wild type IDH2 homodimers display significantly elevated levels of NADPH production compared to that of mutant IDH2 homodimers (Figure 5b). Mutant IDH2 homodimers are unable to generate NADPH in the presence of isocitrate and the NADPH levels observed correlate directly with those of endogenous negative control levels (Figure 5b). However, unlike IDH1 heterodimers, wild type and mutant IDH2 heterodimers are unable to generate NADPH higher than the levels observed for the endogenous control (Figure 5b). This suggests that mutant IDH2 renders wild type IDH2 unable to generate NADPH in the heterodimer since 50% wild type IDH2-VN173 is able to generate significant levels of NADPH (Figure 5b). Together, this data demonstrates that wild type and mutant IDH1 heterodimers are active with respect to their ability to generate NADPH but wild type and mutant IDH2 heterodimers are inactive and do not produce NADPH. This suggests that mutant IDH2, but not mutant IDH1, acts in a dominant negative fashion.

### Wild type and mutant IDH heterodimer enzymatic activity can be altered depending on the predominant dimer in the cell

To determine if the enzymatic activity of IDH wild type and mutant heterodimers is dynamic, we performed a competition-based assay whereby increasing concentrations of HA-tagged wild type IDH1 or IDH2 were co-transfected with wild type and mutant IDH1 or 2 heterodimers, respectively (Figure 5c–d). Basal activity levels of IDH heterodimers replicate the data observed in Figure 5a–b whereby IDH1 heterodimers show enzymatic activity while IDH2 heterodimers do not (Figure 5c–d ‘Open Circle’). However, in both cases, increasing amounts of HA-tagged wild type IDH1 or IDH2 constructs were able to significantly increase the ability of the dimers to generate NADPH up to saturation levels (Figure 5c–d ‘Closed Square’).

### Generation of 2-HG from IDH-Venus Dimers

**Figure 6:**
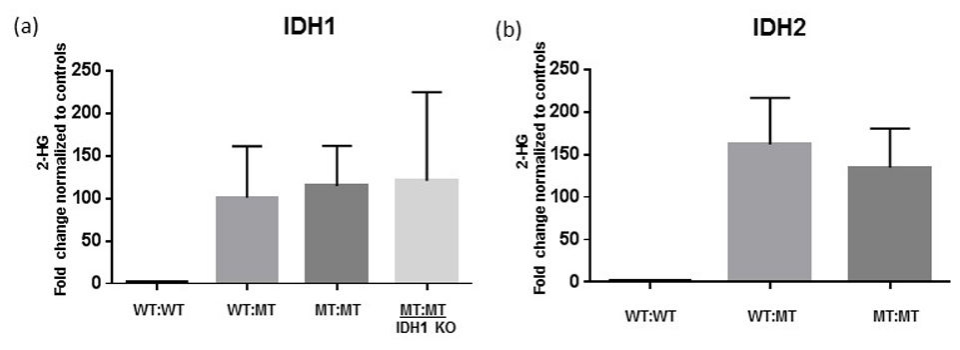
Wild type and mutant IDH heterodimers and mutant homodimers generate 2-HG HEK293FT cells were transfected with the indicated combinations of wild-type and mutant IDH1 (a) or IDH2 (b) BiFC constructs and assessed for their ability to generate 2-HG using GC-MS. Results are representative of three independent experiments. IDH1 KO refers to HEK293FT cells that do not express endogenous IDH1.

The observation that wild type and mutant IDH1 heterodimers generate NADPH but that wild type and mutant IDH2 heterodimers do not, led us to determine the levels of 2-HG produced by IDH homo- and heterodimers. Cellular expression of mutant IDH1 or IDH2 leads to the production of the oncometabolite 2-HG such that elevated levels of 2-HG have been detected in-patients harboring these mutations (8, 9). To assess 2-HG levels in cells expressing IDH1 or IDH2 Venus fusion constructs, a mass spectrometric approach was utilized to compare 2-HG production in cells expressing the indicated IDH-Venus fusion constructs (Figure 6a–b). As expected, wild type IDH homodimers generated low levels of 2-HG (Figure 6a–b) while mutant IDH homodimers generated significantly elevated levels of 2-HG (Figure 6a–b). Both IDH1 and IDH2 wild type and mutant heterodimers were able to generate significantly elevated levels of 2-HG at concentrations similar to that of mutant IDH homodimers (Figure 6a–b). These findings demonstrate that, despite differences in the ability of IDH1 or IDH2 heterodimers to generate NADPH (Figure 5a–b) both are able to generate significant levels of 2-HG (Figure 6a–b). Because HEK293FT cells express IDH endogenously, it is possible that heterodimers may form between endogenous wild type IDH and mutant IDH to contribute to 2-HG production. To evaluate the effect of endogenous IDH1 on 2-HG production, we used CRISPR/CAS9 gene editing to knockout IDH1 expression. Single cell clones were expanded and CRISPR/CAS9 mediated *IDH1* sequence modification was assessed using High Resolution Melt Analysis (Supplementary Figure 2). Clones 5, 7 and 9 demonstrated gene editing by HRMA and showed the least IDH1 protein expression (Supplementary Figure 3a). These clones were propagated to achieve disruption of both IDH1 alleles, which was confirmed by immunoblot (Supplementary Figure 3b) and sequencing. IDH1 knockout (KO) cells were transfected with wild type or mutant IDH1 Venus fusion constructs and 2-HG production was quantified using mass spectrometry (Figure 6a). Loss of endogenous IDH1 had no effect on the ability of mutant IDH homodimers to produce 2-HG, which suggests that the wild type IDH allele is not required in this context.

## Discussion

In this study, we characterize the dimerization and function of wild type and mutant IDH homo- and heterodimers using Bimolecular Fluorescence Complementation (BiFC). BiFC has been used extensively to study protein-protein interactions in living cells and has characterized both known and novel protein-protein interactions (18, 19, 21, 22). We utilized this approach to characterize the interaction between wild type and mutant IDH1 or 2 homo- and heterodimers. The recent observation that IDH mutations have been found in 80% of WHO II–III gliomas and secondary glioblastomas has led to extensive analysis into the role of these mutations in brain tumor etiology. As such, the function of these heterozygous point mutations has been studied extensively by overexpressing mutant IDH *in vitro*. However, the function of wild type and mutant IDH heterodimers has not been assessed. IDH proteins function as dimers and, thus, the heterodimerization between wild type and mutant IDH monomers is likely regulating the cellular function of mutant IDH.

A BiFC-based approach allows for the direct visualization of protein interactions in living cells, and is based on complementation between two non-fluorescent fragments of Venus protein. When these fragments are brought into close proximity, through the interactions of proteins that they’re tethered to, the two fragments fluoresce (23). As such, when the two fragments of Venus are tethered to wild type or mutant IDH, interactions between these monomers will restore fluorescence. We observed similar fluorescent intensities for both IDH homodimer and heterodimer interactions suggesting that wild type and mutant heterodimerization or mutant-mutant homodimerization is as efficient as wild type homodimerization. BiFC-conjugated wild type and mutant IDH homo- and heterodimerization were confirmed by both protein expression and fluorescent microscopy. Subcellular localization was also confirmed with fluorescent microscopy and demonstrated localization of IDH1 to the cytosol and IDH2 to the mitochondria.

IDH catalyzes the oxidative decarboxylation of isocitrate to α-ketoglutarate generating NADPH. Mutations in IDH have been shown to alter the isocitrate binding site such that it inhibits the production of α-ketoglutarate and thus NADPH (7, 8). To measure the activity of BiFC-conjugated IDH wild type and mutant homo- and heterodimers we sought to measure the production of NADPH. Here we observed that, in the case of IDH1, wild type and mutant heterodimers demonstrate physiologically normal activity compared to inactive mutant IDH1 homodimers. Conversely, we found no significant activity difference between wild type and mutant IDH2 heterodimers and mutant IDH2 homodimers. This data suggests that wild type and mutant IDH1 heterodimers are enzymatically active while wild type and mutant IDH2 heterodimers are enzymatically inactive, which demonstrates a fundamental difference between wild type and mutant IDH1 and 2 heterodimers. IDH1 and IDH2 share 70% protein homology (24) and the most common R132H and R172K mutations in IDH1 and IDH2, respectively, are located in the enzymes’ active site (6). These mutations are predicted to have similar effects on their respective IDH proteins based on alignment analysis (25). However, this is the first study to examine wild type and mutant heterodimer activity and, as such, demonstrates a fundamental difference in the activity levels between IDH1 and IDH2 wild type and mutant heterodimers. IDH2 mutations are also significantly less common in glioma than their IDH1 counterparts suggesting that IDH1 mutations may be more advantageous to the tumor cells than IDH2 mutations. Based on our observations, we propose that this could be as a result of the ability of IDH1 wild type and mutant heterodimers to produce NADPH while IDH2 wild type and mutant heterodimers do not. NADPH plays a key role in cellular defense against oxidative damage. Loss of NADPH would have significant catastrophic effects on the cell such that the proliferative advantage of cancer cells may be compromised. The fact that wild type and mutant IDH1 heterodimers are able to maintain physiological NADPH levels suggests that the majority of IDH1 mutations effects may be through 2-HG production.

It has recently been demonstrated that the potential for IDH1 mutations to produce 2-HG is dependent on a retained wild type allele (14). In our studies, we found that 2-HG levels were comparable between wild type and mutant heterodimers and mutant homodimers suggesting that enzymatic activity but not production of 2-HG differs between isoform and/or dimerization partners. To assess whether endogenous IDH1 was responsible for 2-HG production, we used CRISPR gene editing to knockout IDH1 expression. Loss of endogenous IDH1 had no effect on the ability of mutant IDH1 homodimers to produce 2-HG, which suggests that the wild type IDH1 allele is not required in this context. In our studies, we utilized equal amounts of DNA such that the ratio of wild type:mutant IDH1 heterodimers and mutant IDH1 homodimers remained equal. Consequently, we were able to directly quantify the amount of 2-HG produced by equal amounts of wild type and mutant heterodimers and mutant homodimers. Thus, we demonstrate that when the amount of IDH heterodimers and mutant homodimers are equal, 2-HG production is comparable.

We also sought to investigate whether the enzymatic activity of IDH wild type and mutant heterodimers was dynamic such that activity could be restored. We observed that excess wild type IDH1 or IDH2 could restore the enzymatic activity of both IDH1 and IDH2 mutant homodimers as measured by NADPH production in the presence of isocitrate. Together, these data suggest that the dimerization and thus activity of IDH dimers is dynamic such that altering the stoichiometry of the dimers in a cell could rescue the aberrant function of mutant IDH. Targeted inhibition of mutant IDH has the potential to allow for an increase in wild type IDH homodimer formation such that normal cellular activity is restored. We propose that our fluorescent BiFC IDH dimer model could also be used as an approach to screen drugs that prevent and reverse wild type:mutant and/or mutant:mutant dimers. Thus, this model has the potential to be used as an approach for a high-throughput drug screen such that inhibitors of aberrant IDH dimerization can be assessed based on loss of fluorescence. Whether mutant IDH, or products of its activity, can be specifically targeted for therapeutic intervention in glioma patients has yet to be determined. If mutant IDH is required for tumor formation, progression or maintenance, it is feasible that targeting the dimerization of IDH may be a viable target for therapeutic intervention.

## Supplementary Material

SFigure 1 Supplementary Figure 1. Control BiFC combinations demonstrate fluorescent complex formation only with complementary binding partners HEK293FT cells were co-transfected with the indicated constructs and assessed for BiFC fluorescence 24 h post-transfection. Images are representative of two independent experiments.

SFigure 2 Supplementary Figure 2. HRMA detects the presence of mutant IDH1 alleles (a) Left panel: Normalized melting curves show DNA amplified from the wild type *IDH1^+/+^* genome (green curves), which comprise a homogeneous population of duplexes with a single Tm. In contrast, re-annealed amplicons derived from the *IDH1^Δ/+^* genomes of clones 1–5 (gray, red, and blue curves as labeled) are composed of multiple duplex populations, which display distinct Tms. Right panel: difference fluorescence curves for clones 1–5. (b) Left panel: Normalized melting curves show DNA amplified from the *IDH1^+/+^* genome (gray curves), which comprise a homogeneous population of duplexes with a single Tm. DNA amplified from the *IDH1^Δ/+^* clone 10 genome (turquoise curves) is similar to the wild type genome indicating that gene editing did not occur. In contrast, re-annealed amplicons derived from the *IDH1^Δ/+^* genomes of clones 6–9 (blue, orange, green, and red curves as labeled) are composed of multiple duplex populations, which display distinct Tms. Right panel: difference fluorescence curves for clones 6–10.

SFigure 3 Supplementary Figure 3. Immunoblotting demonstrates Cas9 expression and IDH1 knockout a) Immunoblot analysis of Cas9, IDH1, and GAPDH from whole cell lysates from 10 IDH1 CRISPR/CAS9 HEK293FT clones compared to whole cell lysate from uninfected wild type (WT) HEK293FT cells. (b) Clones 5, 7 and 9 were further propagated to achieve complete knockout of IDH1. GAPDH was used as a loading control.
**Supplementary Table 1. Primers used for asymmetric PCR.** List of primers used for generating the indicated IDH-Venus construct.

## Abbreviations

α-KG: *alpha-ketoglutarate*
2HG: 2-Hydroxyglutarate
BiFC: bimolecular fluorescence complementation
CAS: CRISPR-associated protein 9
CRIPSR: clustered regularly interspaced short palindromic repeats
DMEM: Dulbecco’s modified eagle medium
ECL: enhanced chemiluminescence
GAPDH: glyceraldehyde 3-phosphate dehydrogenase
GBM: *glioblastoma* multiforme
gRNA: guide RNA
HA: human influenza hemagglutinin
HEK293T: human embryonic kidney 293T
HRMA: high resolution melt analysis
IDH: isocitrate dehydrogenase
KO: knock out
NADPH: nicotinamide adenine dinucleotide phosphate
RalA: Ras-related protein
RalBP1: RalA binding protein
TEV: tobacco Etch Virus
VC: C-terminal residues (155-238) of Venus
VN: N-terminal residues (155-238) of Venus
WHO: world health organization
