# Supplementary figures and images for: In vitro visualization and characterization of wild type and mutant IDH homo- and heterodimers using Bimolecular Fluorescence Complementation
Source: Cancer Res Front. Author manuscript; Available in PMC 2017 May 4. (PMC5417691; doi:10.17980/2016.311)

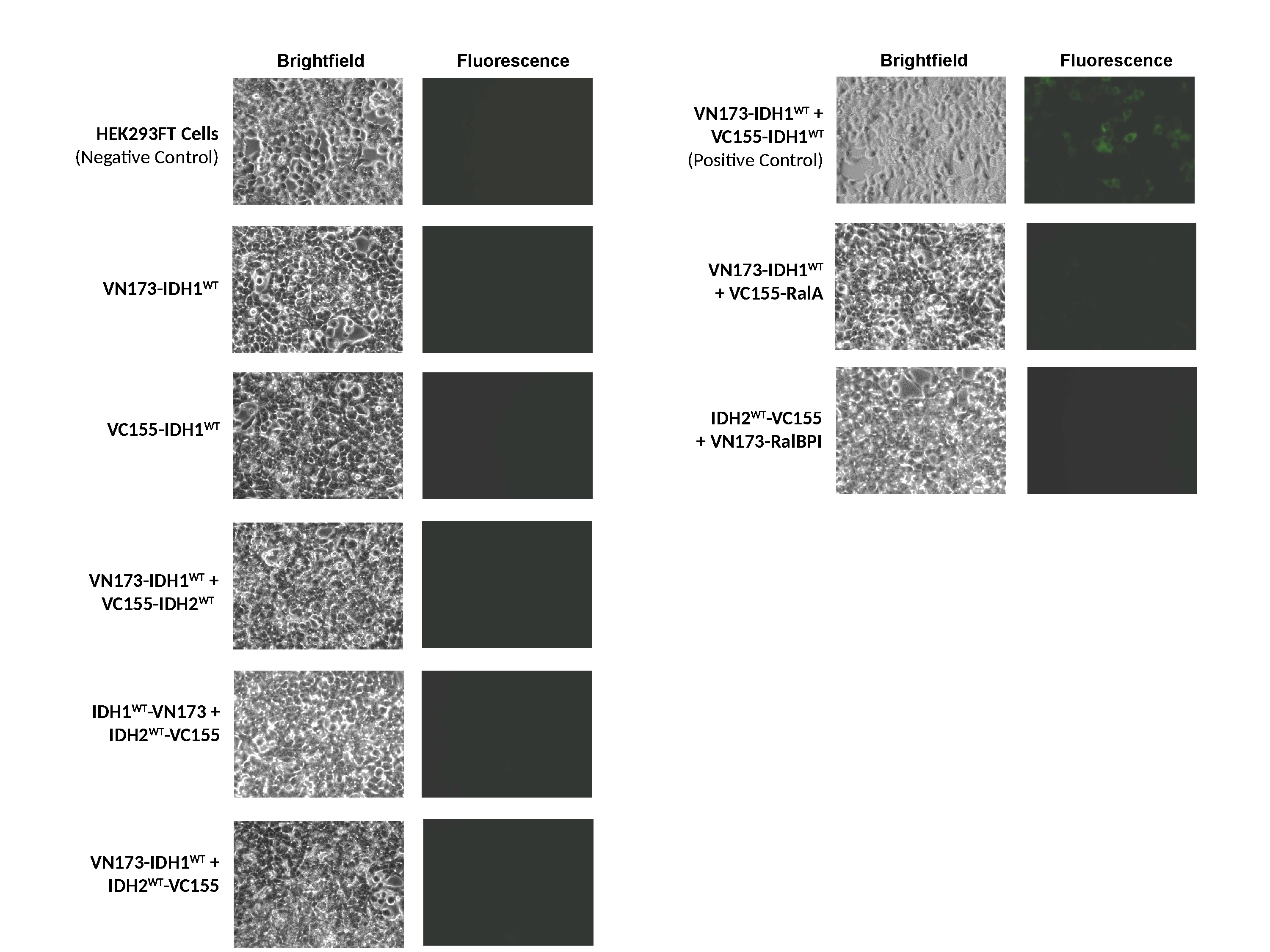

Supplement: SFigure 1 — HEK293FT cells were co-transfected with the indicated constructs and assessed for BiFC fluorescence 24 h post-transfection. Images are representative of two independent experiments. [file NIHMS820350-supplement-SFigure_1.tiff]

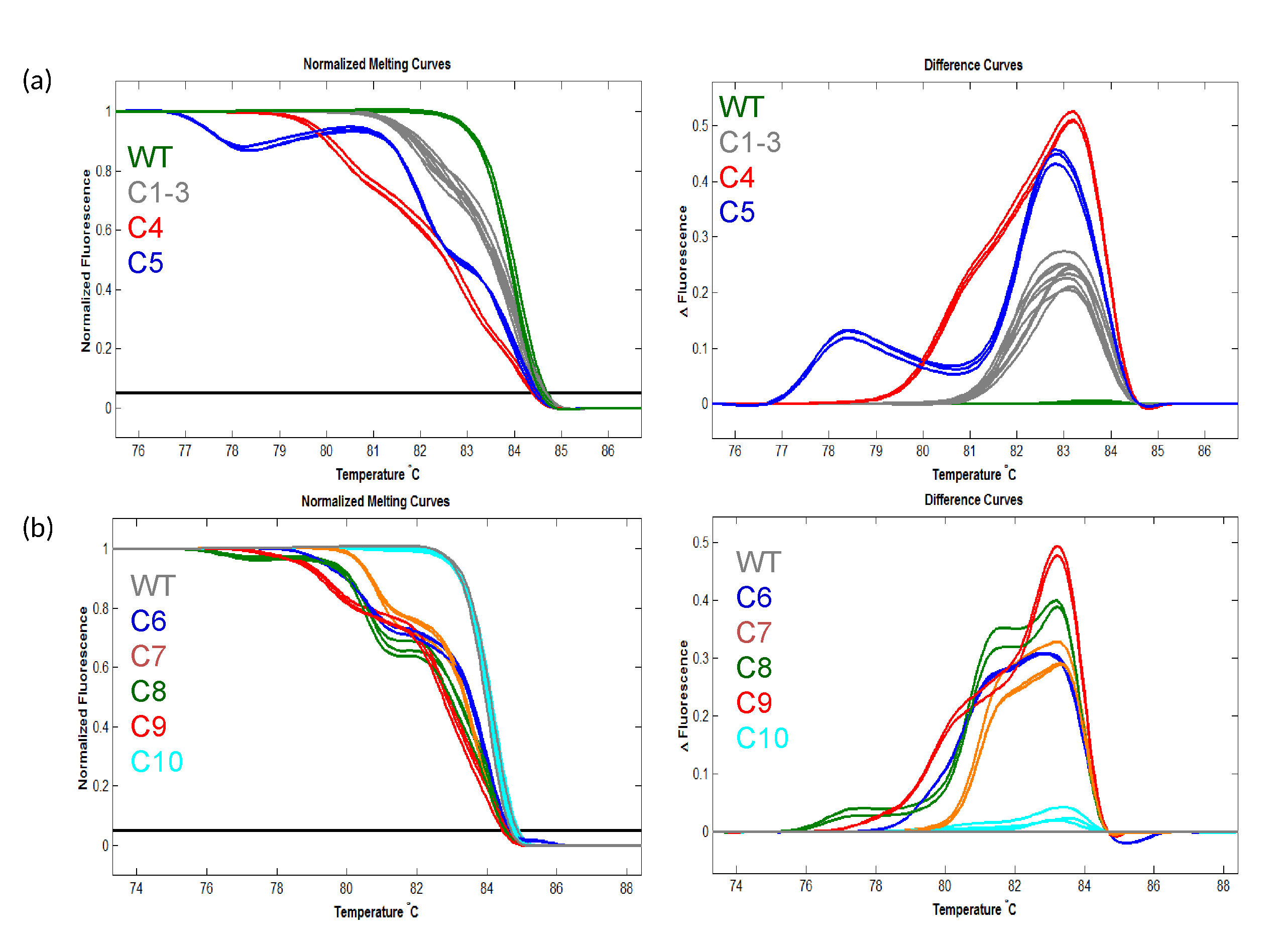

Supplement: SFigure 2 — (a) Left panel: Normalized melting curves show DNA amplified from the wild type IDH1+/+ genome (green curves), which comprise a homogeneous population of duplexes with a single Tm. In contrast, re-annealed amplicons derived from the IDH1Δ/+ genomes of clones 1–5 (gray, red, and blue curves as labeled) are composed of multiple duplex populations, which display distinct Tms. Right panel: difference fluorescence curves for clones 1–5. (b) Left panel: Normalized melting curves show DNA amplified from the IDH1+/+ genome (gray curves), which comprise a homogeneous population of duplexes with a single Tm. DNA amplified from the IDH1Δ/+ clone 10 genome (turquoise curves) is similar to the wild type genome indicating that gene editing did not occur. In contrast, re-annealed amplicons derived from the IDH1Δ/+ genomes of clones 6–9 (blue, orange, green, and red curves as labeled) are composed of multiple duplex populations, which display distinct Tms. Right panel: difference fluorescence curves for clones 6–10. [file NIHMS820350-supplement-SFigure_2.tiff]

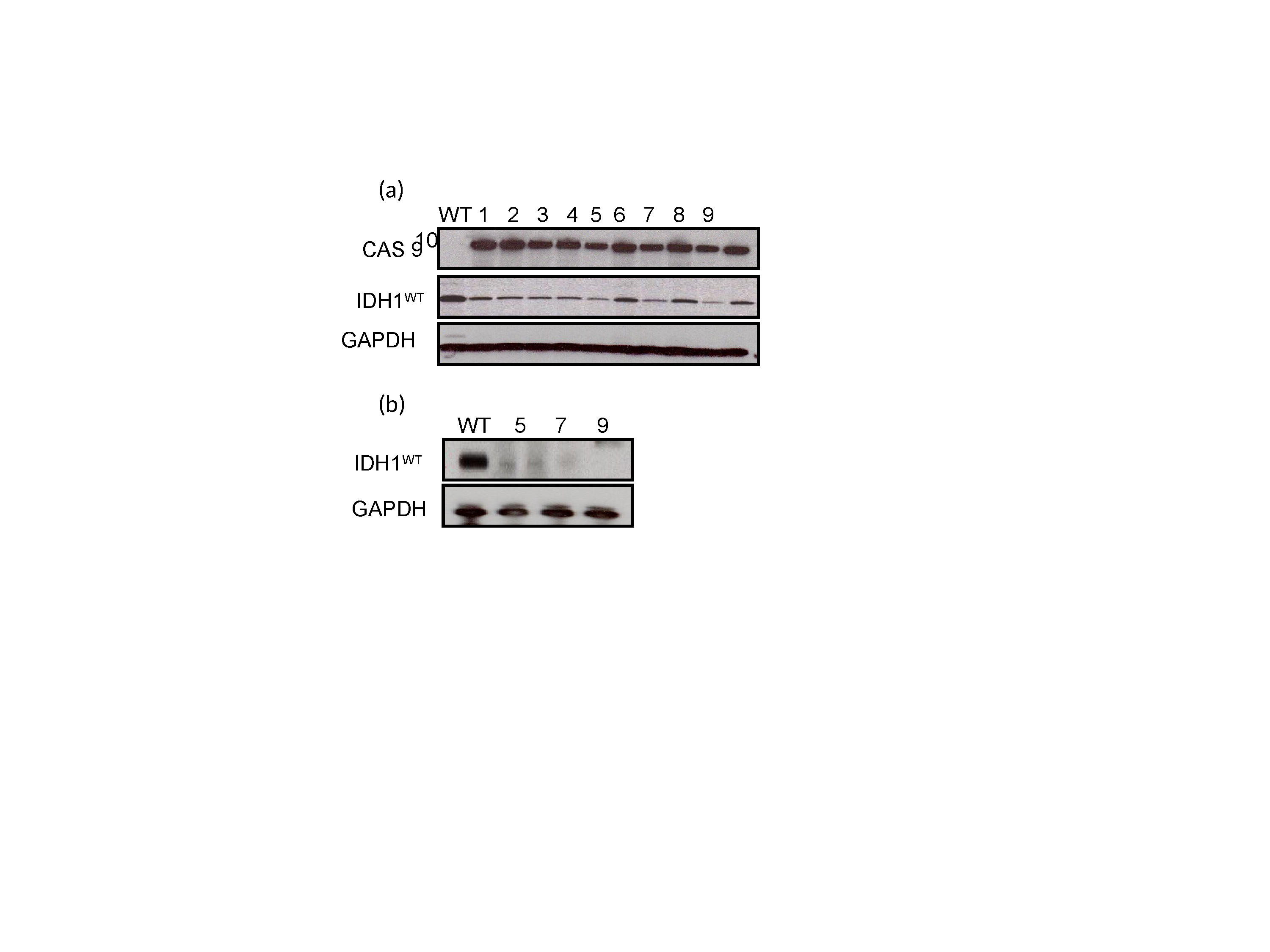

Supplement: SFigure 3 — a) Immunoblot analysis of Cas9, IDH1, and GAPDH from whole cell lysates from 10 IDH1 CRISPR/CAS9 HEK293FT clones compared to whole cell lysate from uninfected wild type (WT) HEK293FT cells. (b) Clones 5, 7 and 9 were further propagated to achieve complete knockout of IDH1. GAPDH was used as a loading control. Supplementary Table 1. Primers used for asymmetric PCR. List of primers used for generating the indicated IDH-Venus construct. [file NIHMS820350-supplement-SFigure_3.tiff]
